# Robotic Magnetic Navigation Versus Manual Catheter Ablation for Premature Ventricular Contractions: A Single-center Retrospective Cohort Study

**DOI:** 10.19102/icrm.2026.17062

**Published:** 2026-06-15

**Authors:** Moez Alnazeer, Jerry Fan, Thao Giang, Christopher Euell, Jessica Lee, Bright Izekor, Christopher Perez, Syed Zamin, Kaylee Pascarella, Javier Banchs, Gregory Olsovsky

**Affiliations:** 1 Division of Cardiology, Baylor College of Medicine, Baylor Scott & White Medical Center—Temple, Temple, TX, USA; 2 Department of Internal Medicine, Baylor College of Medicine, Baylor Scott & White Medical Center—Temple, Temple, TX, USA; 3 Department of Statistics, Baylor University, Waco, TX, USA

**Keywords:** Anti-arrhythmic drugs, catheter ablation, premature ventricular contractions, robotic magnetic navigation, Stereotaxis

## Abstract

Robotic magnetic navigation (RMN) offers potential advantages for premature ventricular contraction (PVC) ablation, including improved catheter stability and reduced operator radiation exposure, but comparative data versus conventional manual ablation for PVCs are limited. The objective of this study was to compare procedural metrics and very short-term clinical outcomes—specifically, 1-month symptom status, class I/III anti-arrhythmic drug (AAD) use, and periprocedural complications—between RMN-guided and manual PVC ablation in a single-center experience. We performed a retrospective cohort study of consecutive patients undergoing PVC ablation at a single center between 2019 and 2023. Patients were categorized by technique: conventional manual ablation or RMN using the Niobe ES system (Stereotaxis, St. Louis, MO, USA). Of 93 unique patients, 74 initially underwent RMN and 19 underwent manual ablation. Seven RMN cases requiring intraprocedural conversion to manual ablation were excluded from the primary comparison, yielding a per-protocol analytic cohort of 86 patients (67 RMN and 19 manual). Baseline characteristics, including age, left ventricular ejection fraction (LVEF), comorbidities, pre-procedure PVC burden, and class I/III AAD use, were compared between groups. The primary endpoints were total procedure time and fluoroscopy time, while secondary endpoints included 1-month symptom resolution, post-procedure class I/III AAD use, and periprocedural complications. An intention-to-treat sensitivity analysis grouped converted cases with the RMN-initial cohort. Baseline characteristics were similar between the RMN and manual cohorts (mean age, 62.8 ± 13.1 vs. 62.2 ± 12.8 years; LVEF, 46.2% ± 13.5% vs. 50.8% ± 14.8%; left ventricular [LV] systolic dysfunction [LVEF < 50%], 44.8% vs. 31.6%; all *P* > .2). Mean procedure times were comparable (212.3 ± 67.3 vs. 218.5 ± 74.7 min; *P* = .747), as were fluoroscopy times (12.3 ± 7.2 vs. 12.4 ± 9.0 min; *P* = .968). At 1 month, symptom resolution was documented in 74.6% (47/63) of RMN patients and 62.5% (10/16) of manual patients (*P* = .514). Class I/III AAD use decreased from 89.6% to 61.2% in the RMN group and from 89.5% to 68.4% in the manual group (net change, −28.4% vs. −21.1%; *P* value for post-procedure comparison = .759) without a statistically significant between-group difference. Periprocedural complications did not differ significantly (3.0% RMN vs. 5.3% manual; *P* = .532): one tamponade occurred in the manual group, and one pericardial effusion and one pseudoaneurysm occurred in the RMN group. In this single-center retrospective cohort, RMN-guided PVC ablation achieved procedure times, fluoroscopy times, very short-term symptom outcomes, and complication rates comparable to conventional manual ablation, with substantial reductions in class I/III AAD use in both groups at 1 month. Findings were similar in an intention-to-treat sensitivity analysis that grouped converted cases with the RMN-initial cohort. Because post-ablation PVC burden and follow-up LV function were not systematically assessed, these results should be interpreted as hypothesis-generating and limited to procedural efficiency and early, largely subjective outcomes.

## Introduction

Premature ventricular contractions (PVCs) are common ectopic beats that interrupt normal sinus rhythm. In many individuals with structurally normal hearts, infrequent PVCs are benign and require no specific therapy. However, frequent or highly symptomatic PVCs, often operationally defined as a burden >10%–15% of total beats, have been implicated in the development of left ventricular (LV) dysfunction and a reversible form of PVC-induced cardiomyopathy.^1–3^ Suppression of PVCs in such patients can lead to improvement or normalization of LV ejection fraction (LVEF), highlighting PVCs as both a marker and a potential driver of ventricular dysfunction.^4–9^

Initial management typically relies on medical therapy with β-blockers or non-dihydropyridine calcium channel blockers, but these agents often have a modest impact on PVC burden and may be limited by side effects.^10,11^ Class I and III anti-arrhythmic drugs (AADs) can be more effective in suppressing PVCs but carry the risk of pro-arrhythmia and other toxicities, making long-term therapy undesirable for many patients.^10,11^ Consequently, catheter ablation has become an established treatment option for patients with frequent or symptomatic PVCs, especially when there is concern for PVC-induced cardiomyopathy or when medical therapy fails or is not tolerated.^4–8,10,11^ Contemporary series report high acute success rates and meaningful reductions in PVC burden after ablation, with associated improvements in LV function in susceptible patients.^4–9^

Conventional manual PVC ablation is typically performed using three-dimensional electroanatomic mapping with fluoroscopy used as needed for catheter positioning, although fluoroless workflows are increasingly feasible in contemporary practice.^10,11^ This approach is effective for many outflow tract and fascicular PVCs but can be technically challenging in anatomically complex or difficult-to-reach locations, such as papillary muscles, intramural septal sites, the LV summit, or epicardial and venous structures near the coronary arteries.^9–11^ In these settings, catheter stability, contact, and reach may be suboptimal, potentially limiting ablation efficacy or increasing procedure time and fluoroscopy exposure.^9–11^

Robotic magnetic navigation (RMN) systems, such as the Niobe ES system (Stereotaxis, St. Louis, MO, USA), were developed to address some of these challenges.^12–16^ RMN uses external magnets to steer a soft-tipped, magnetically responsive catheter within the heart, controlled by the operator from a remote workstation.^12–14^ The technology offers precise and highly flexible catheter manipulation, which may facilitate stable contact in complex ventricular anatomy, while substantially reducing operator radiation exposure and physical strain.^12–18^ Prior studies in supraventricular and ventricular tachycardia ablation have demonstrated that RMN can achieve comparable acute success and safety relative to manual ablation, often with reduced fluoroscopy times and potential advantages in difficult substrates.^12–18^

Evidence specifically comparing RMN with manual techniques for PVC ablation, however, remains limited. Available data come largely from single-center retrospective series and small prospective cohorts of ventricular arrhythmias, and few studies have focused on short-term clinical outcomes such as symptom relief and post-ablation AAD use across a broad spectrum of PVC locations and underlying structural heart disease.^19–21^ In particular, whether RMN provides similar procedural efficiency and safety while facilitating reductions in class I/III AAD therapy in routine clinical practice is not well defined.^19–21^

In this context, we performed a single-center retrospective cohort study comparing RMN-guided PVC ablation with conventional manual catheter ablation. Our primary objective was to compare procedural efficiency, as reflected by total procedure time and fluoroscopy time, between techniques. The secondary objectives were to evaluate very short-term clinical outcomes, including 1-month clinician-documented symptom resolution, post-procedure class I/III AAD use, and periprocedural complications, and to explore these outcomes across PVC locations and in patients with and without LVEF <50%. We did not systematically collect post-ablation PVC burden or follow-up LV function; therefore, the study was not designed to compare long-term arrhythmia control or ventricular recovery. We hypothesized that RMN-guided PVC ablation would demonstrate procedural metrics and early clinical outcomes comparable to manual ablation, with the potential to facilitate de-escalation of class I/III AAD therapy.

## Methods

### Study design and population

We conducted a retrospective single-center cohort study of patients who underwent catheter ablation for PVCs at Baylor Scott & White Medical Center—Temple between January 2019 and December 2023. Patients were identified from electrophysiology laboratory procedure logs and electronic medical records.

Patients were eligible if they had symptomatic PVCs or suspected PVC-induced cardiomyopathy with a high PVC burden—typically >10%–15% of total beats and often >10,000 PVCs per 24 h on ambulatory monitoring—and were referred for catheter ablation after inadequate response or intolerance to medical therapy. There was no rigid protocol-mandated numeric cutoff; the decision to proceed with ablation was made at the discretion of the treating electrophysiologist, integrating symptom severity, quantified PVC burden, and clinical suspicion for PVC-induced cardiomyopathy. Both patients with presumed idiopathic PVCs and those with LV systolic dysfunction were included, provided that suppression of PVCs was the primary indication for ablation. For this analysis, LV systolic dysfunction was defined pragmatically as LVEF < 50%.

A total of 93 unique patients met these criteria and formed the source population. Of these, 74 patients initially underwent ablation using an RMN system (Stereotaxis), while 19 underwent conventional manual catheter ablation. Seven RMN cases required intraprocedural conversion to manual ablation. These hybrid procedures were excluded from the primary comparative analysis to preserve a clear distinction between purely robotic and purely manual techniques, yielding a final per-protocol analytic cohort of 86 patients (67 RMN and 19 manual). In addition, an intention-to-treat (ITT) sensitivity analysis grouped all 74 RMN-initial cases, including the seven conversions, together and compared them with the 19 manual-initial cases.

### Ablation techniques

***Manual catheter ablation.*** Manual ablation procedures were performed using contemporary three-dimensional electroanatomic mapping systems (CARTO™ 3 [J&J MedTech, New Brunswick, NJ, USA] or EnSite™ Precision [Abbott, Chicago, IL, USA]) at the discretion of the operator. Mapping and ablation were performed with conventional steerable open-irrigated radiofrequency catheters. Catheter manipulation was performed at the bedside under fluoroscopic and electroanatomic guidance. Activation mapping and/or pace-mapping were used to localize the PVC origin in the chamber of interest according to standard practice. Once the presumed focus was identified, radiofrequency energy was delivered in a point-by-point fashion to eliminate the ectopic source.

***Robotic magnetic navigation.*** RMN-guided procedures were performed using the Stereotaxis remote magnetic navigation system. A magnetically responsive, flexible ablation catheter was introduced percutaneously and steered within the heart by adjusting external magnetic field vectors from a remote workstation. Catheter advancement and withdrawal were performed mechanically at the table (eg, via automated drive or assistant), while electroanatomic mapping was performed using the same mapping platforms as in manual cases. After localization of the PVC focus with activation and/or pace-mapping, radiofrequency energy was delivered through the magnetic catheter.

In both groups, vascular access was obtained via standard femoral venous and, when needed, arterial approaches. Most procedures were performed under general anesthesia. For left-sided ablations, systemic anticoagulation with intravenous heparin was administered to maintain an activated clotting time of >300 s. Radiofrequency applications were delivered using temperature-controlled, power-limited settings (typically 30–50 W, titrated to the site and catheter), with irrigation according to catheter specifications. In general, RMN was preferentially selected for anatomically complex or difficult-to-stabilize targets (eg, coronary sinus [CS]/epicardial-adjacent sites, papillary muscles, LV summit-adjacent regions, or other challenging LV geometries), where directional control and catheter stability were felt to be advantageous. Manual ablation was more commonly used when RMN was unavailable or when operator judgment favored conventional manipulation based on anticipated workflow or catheter support.

For the primary comparison, patients who required conversion from RMN to manual ablation during the same procedure were analyzed separately and not included in either pure RMN or pure manual cohorts. Thus, all primary RMN versus manual analyses reflect a per-protocol comparison, with converted cases reported descriptively as a distinct conversion cohort.

### Data collection and variables

Clinical and procedural data were abstracted from a de-identified institutional dataset and the corresponding medical records.

Baseline variables included:

- Age (years)
- LVEF (%)
- Presence of LV systolic dysfunction (LVEF < 50%)
- Hypertension
- Obstructive sleep apnea
- Pre-procedure PVC burden (%)
- Pre-procedure use of class I or III AADs

Procedural metrics included:

- Total procedure time (min)
- Fluoroscopy time (min)

The PVC anatomical location was categorized for exploratory analyses as right ventricular outflow tract (RVOT), left ventricular outflow tract (LVOT), CS/epicardial, papillary muscle, combined LVOT/RVOT, other LV free wall, fascicular/septal, or mitral annulus.

Short-term clinical outcomes at approximately 1 month were obtained from follow-up clinic documentation or structured telephone encounters where applicable and included:

- *Symptom resolution:* Documented complete resolution of PVC-related symptoms (eg, palpitations or awareness of ectopy) at approximately 1-month follow-up. Patients without clear documentation were coded as missing for this endpoint, and denominators for symptom resolution reflect available data. Analyses of symptom resolution therefore used a complete-case approach, and no imputation was performed for missing values.
- *AAD use:* Presence or absence of class I or III AAD therapy at baseline and at 1-month follow-up, summarized as the proportion of patients on therapy at each time point and the net change in use.
- *Periprocedural complications:* Major complications recorded in the dataset, including pericardial tamponade; pericardial effusion; and vascular complications, such as pseudoaneurysm.

### Endpoints

The primary endpoints were:

- Total procedure time (min)
- Fluoroscopy time (min)

The secondary endpoints focused on very short-term clinical outcomes and included:

- Symptom resolution at approximately 1 month (documented complete resolution of PVC-related symptoms in clinic notes or structured telephone follow-up)
- Post-procedure use of class I/III AADs at 1 month and net change in AAD use from baseline
- Periprocedural complications

Post-ablation PVC burden (eg, 24-h monitoring) and follow-up LV function were not routinely collected in this retrospective dataset and were therefore not analyzed. Exploratory analyses evaluated procedural metrics and symptom outcomes stratified by PVC location and by the presence versus absence of LVEF < 50%. These subgroup analyses were prespecified as descriptive and hypothesis-generating.

### Statistical analysis

All statistical analyses for this project were performed using R version 4.4.2 (R Foundation for Statistical Computing, Vienna, Austria).

Continuous variables are reported as mean ± standard deviation values (and medians with ranges where informative). Categorical variables are summarized as counts and percentages.

Between-group comparisons (RMN vs. manual) were conducted using two-sample *t* tests for continuous variables or Wilcoxon rank-sum tests when distributional assumptions were not met, along with chi-squared tests for categorical variables or Fisher’s exact tests when expected cell counts were small. Comparisons across multiple PVC location strata employed Kruskal–Wallis tests for continuous variables and Fisher’s exact tests for categorical variables.

All hypothesis tests were two-sided, with *P* < .05 considered statistically significant. The sample size was determined by the number of eligible cases during the study period; no formal a priori sample size or power calculation was performed. Given the modest overall cohort and the small manual ablation group, the study was anticipated to be underpowered to detect small-to-moderate between-group differences. No multivariable modeling or adjustment for baseline covariates was performed; all comparisons are unadjusted and intended to be exploratory. Subgroup and location-based analyses were prespecified as descriptive and hypothesis-generating rather than confirmatory. Changes in class I/III AAD use from baseline to 1 month within each group were summarized descriptively; no formal paired tests for pre–post change or interaction between time and ablation strategy were performed.

As a sensitivity analysis, we also performed an ITT comparison based on the initial ablation strategy. For this ITT analysis, all patients first assigned to RMN, including those who required intraprocedural conversion to manual ablation, were analyzed in an RMN-initial group (n = 74) and were compared with patients initially assigned to manual ablation (n = 19). The same endpoints as in the primary per-protocol analysis (procedure time, fluoroscopy time, 1-month symptom resolution, class I/III AAD use, and periprocedural complications) were evaluated. Between-group comparisons used the same testing framework as described already.

An additional sensitivity analysis excluded CS/epicardial cases (which were performed exclusively with RMN) from procedural time comparisons to assess potential bias from location-driven complexity.

### Ethical considerations

The study protocol was approved by the institutional review board with a waiver of individual informed consent due to the retrospective design and use of de-identified data.

## Results

### Study population

**Figure 1:**
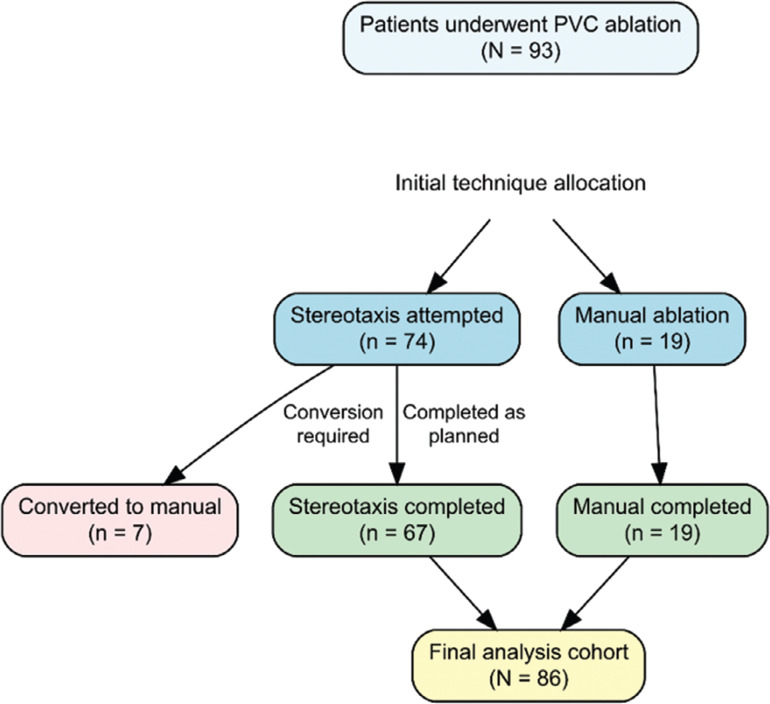
Patient flow diagram. *Abbreviation:* PVC, premature ventricular contraction.

Ninety-three patients underwent PVC ablation during the study period. Of these, 74 procedures were initially performed with RMN and 19 were performed with conventional manual catheter manipulation. Seven RMN cases required intraprocedural conversion to manual ablation and were excluded from the primary comparative analysis, leaving 86 patients in the final cohort (**Figure 1**): 67 who completed RMN-guided ablation and 19 who underwent manual ablation. Thus, the RMN versus manual results presented below reflect outcomes among procedures completed with the originally selected technique (per-protocol analysis).

### Baseline characteristics

Baseline characteristics are summarized in **Table 1**. The mean age of the cohort was 62.7 ± 12.9 years and was similar between the manual and RMN groups (62.2 ± 12.8 vs. 62.8 ± 13.1 years; *P* = .853). The mean LVEF was 47.3% ± 13.8% overall, with no significant difference between the manual and RMN cohorts (50.8% ± 14.8% vs. 46.2% ± 13.5%; *P* = .234). LV systolic dysfunction (LVEF < 50%) was present in 41.9% of patients (36/86), including 31.6% (6/19) in the manual group and 44.8% (30/67) in the RMN group (*P* = .331). The prevalence of hypertension (63.2% vs. 62.7%; *P* > .99) and that of obstructive sleep apnea (31.6% vs. 32.8%; *P* > .99) did not differ between manual and RMN patients. The pre-procedure PVC burden was also comparable (19.1% ± 9.5% vs. 20.6% ± 11.5%; *P* = .624), consistent with a high-burden referral population in both cohorts. Class I/III AAD use at baseline was high in both cohorts (89.5% in manual and 89.6% in RMN; *P* > .99). Collectively, these findings suggest broadly similar baseline characteristics between groups for comparative analysis.

**Table 1:** Baseline Characteristics of the Study Population

Characteristic	Total (n = 86)	Manual (n = 19)	RMN (n = 67)	*P* Value
Age, years	62.7 ± 12.9	62.2 ± 12.8	62.8 ± 13.1	.853
LVEF, %	47.3 ± 13.8	50.8 ± 14.8	46.2 ± 13.5	.234
LV systolic dysfunction (LVEF < 50%)	36 (41.9%)	6 (31.6%)	30 (44.8%)	.331
Hypertension	54 (62.8%)	12 (63.2%)	42 (62.7%)	1.000
Obstructive sleep apnea	28 (32.6%)	6 (31.6%)	22 (32.8%)	1.000
Pre-procedure PVC burden, %	20.3 ± 11.1	19.1 ± 9.5	20.6 ± 11.5	.624
Pre-procedure AAD use (class I/III)	77 (89.5%)	17 (89.5%)	60 (89.6%)	1.000
PVC location				.160
RVOT	22 (25.6%)	7 (36.8%)	15 (22.4%)	
LVOT	16 (18.6%)	3 (15.8%)	13 (19.4%)	
CS/epicardial	15 (17.4%)	0 (0.0%)	15 (22.4%)	
Papillary muscle	10 (11.6%)	2 (10.5%)	8 (11.9%)	
LVOT/RVOT	7 (8.1%)	2 (10.5%)	5 (7.5%)	
Other LV free wall	6 (7.0%)	3 (15.8%)	3 (4.5%)	
Fascicular/septal	5 (5.8%)	1 (5.3%)	4 (6.0%)	
MV annulus	5 (5.8%)	1 (5.3%)	4 (6.0%)	

*Abbreviations:* AAD, anti-arrhythmic drug; CS, coronary sinus; LV, left ventricular; LVEF, left ventricular ejection fraction; LVOT, left ventricular outflow tract; MV, mitral valve; PVC, premature ventricular contraction; RMN, robotic magnetic navigation; RVOT, right ventricular outflow tract. There were no statistically significant differences between the manual and robotic groups in age, LVEF, prevalence of structural heart disease, comorbidities (hypertension, obstructive sleep apnea), pre-procedure PVC burden, or baseline AAD use, indicating well-balanced groups for comparison.

### Procedural metrics

**Figure 2:**
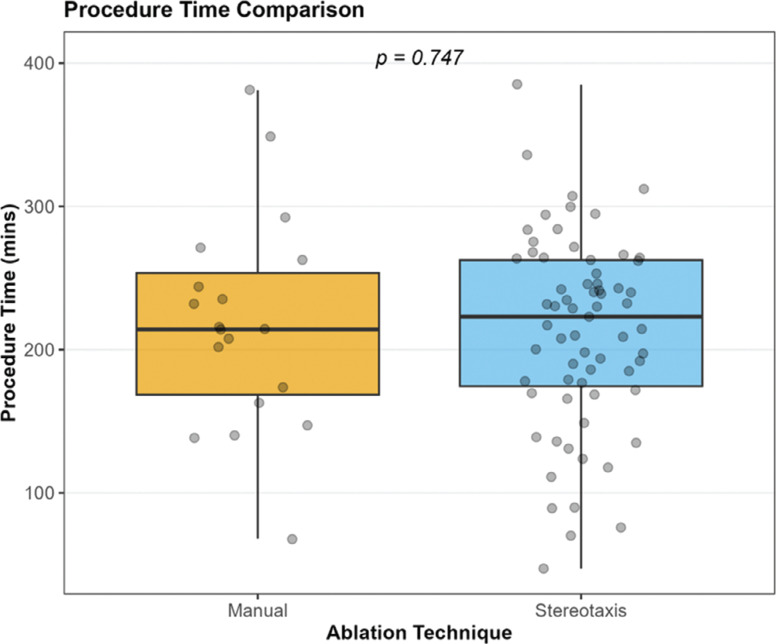
Procedure time comparison. Distribution of procedure times for manual versus robotic ablation. Box plots show median (center line), interquartile range (box), and individual data points (dots). No significant difference was observed between the groups (*P* = .747).

Procedural metrics are shown in **Table 2**. The mean total procedure time was similar between manual and RMN ablation (218.5 ± 74.7 vs. 212.3 ± 67.3 min; *P* = .747) (**Figure 2**). Median procedure times were likewise comparable (214.0 vs. 223.0 min), with broad but overlapping ranges in both groups (68–381 min for manual vs. 47–385 min for RMN).

**Table 2:** Procedural Metrics

Metric	Manual (n = 19)	RMN (n = 67)	*P* Value
Procedure time, min			
Mean ± SD	218.5 ± 74.7	212.3 ± 67.3	.747
Median	214.0	223.0	
Min	68.0	47.0	
Max	381.0	385.0	
Fluoroscopy time, min			
Mean ± SD	12.4 ± 9.0	12.3 ± 7.2	.968
Median	10.5	11.0	
Min	2.1	0.6	
Max	40.9	49.5	

*Abbreviations:* RMN, robotic magnetic navigation; SD, standard deviation. This table compares procedural times between manual and robotic ablation techniques. Mean procedure times were similar between groups (218.5 min for manual vs. 212.3 min for robotic, *P* = .747). Fluoroscopy times were also comparable (12.4 min for manual vs. 12.3 min for robotic, *P* = .968). Neither procedural metric showed statistically significant differences between the techniques.

**Figure 3:**
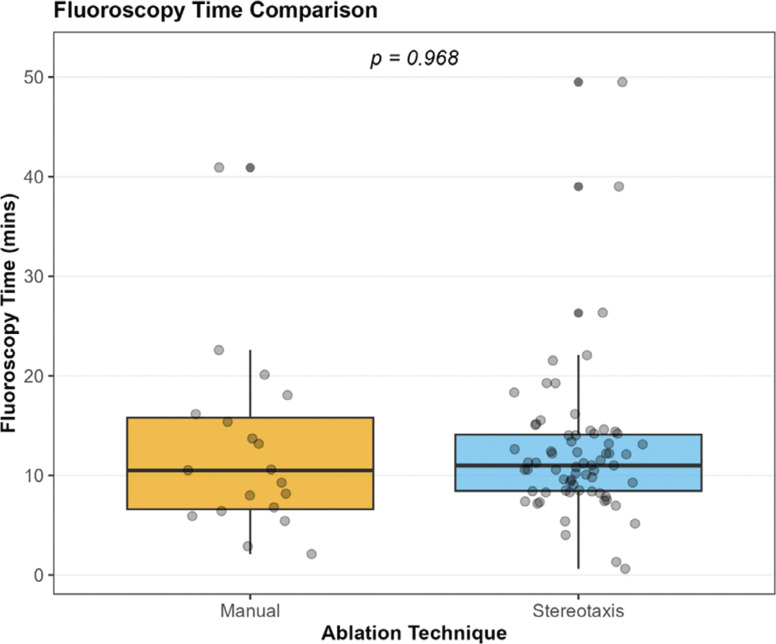
Fluoroscopy time comparison. Distribution of fluoroscopy times for manual versus robotic ablation. Box plots show median (center line), interquartile range (box), and individual data points (dots). No significant difference was observed between the groups (*P* = .968).

The mean fluoroscopy time did not differ between strategies (12.4 ± 9.0 min for manual vs. 12.3 ± 7.2 min for RMN; *P* = .968) (**Figure 3**), with similar medians (10.5 vs. 11.0 min) and ranges (2.1–40.9 vs. 0.6–49.5 min).

We performed sensitivity analyses excluding CS/epicardial cases (n = 15), which occurred exclusively in the RMN group and are typically more time-intensive. Findings were unchanged from the primary analysis. In the as-treated comparison, procedure time (*P* = .550) and fluoroscopy time (*P* = .840) did not differ significantly between techniques; results were similar in the ITT analysis (procedure time, *P* = .751; fluoroscopy time, *P* = .838). Within LV systolic dysfunction subgroups, procedure times also did not differ significantly between techniques (*P* = .466 with and *P* = .978 without LV systolic dysfunction) **(Table S1)**.

**Table S1:** Sensitivity Analysis of Procedural Metrics Excluding Coronary Sinus/Epicardial Cases

Analysis	Manual	RMN	*P* Value
As-treated analysis	(n = 19)	(n = 52^a^)	
Procedure time, min	218.5 ± 74.7	203.6 ± 69.9	.550
Fluoroscopy time, min	12.4 ± 9.0	12.0 ± 7.7	.840
ITT analysis	(n = 19)	(n = 57^b^)	
Procedure time, min	218.5 ± 74.7	208.8 ± 70.7	.751
Fluoroscopy time, min	12.4 ± 9.0	12.4 ± 8.9	.838
LV systolic dysfunction status			
No LV systolic dysfunction	(n = 13)	(n = 29^a^)	
Procedure time, min	215.2 ± 55.4	208.4 ± 69.6	.978
	(n = 6)	(n = 21^a^)	
Procedure time, min	225.7 ± 112.6	198.9 ± 72.3	.466

*Abbreviations:* CS, coronary sinus; HD, heart disease; ITT, intention to treat; LVEF, left ventricular ejection fraction; RMN, remote magnetic navigation; SD, standard deviation. ^a^As-treated: the RMN group excludes CS/epicardial cases (n = 15 from overall, distributed across subgroups). ^b^ITT: the RMN-initial group excludes CS/epicardial cases (n = 15 total). Values are shown as mean ± SD. CS/epicardial cases performed exclusively with RMN were excluded to address potential bias from inherently time-consuming procedures. *P* values from the Wilcoxon rank-sum test are given. LV systolic dysfunction was defined as LVEF < 50%.

### Clinical outcomes at 1 month

Clinical outcomes are summarized in **Table 3**. Symptom resolution at approximately 1 month was assessable in 79 of 86 patients (92%). Overall, 57 of 79 patients (72.2%) reported complete resolution of PVC-related symptoms. Symptom resolution occurred in 62.5% (10/16) of manual patients and 74.6% (47/63) of RMN patients (*P* = .514), indicating no statistically significant difference between techniques.

**Table 3:** Summary of Clinical Outcomes at 1-month Follow-up

Outcome	Total (n = 86)	Manual (n = 19)	RMN (n = 67)	*P* Value
Symptom resolution at 1 month	57/79 (72.2%)	10/16 (62.5%)	47/63 (74.6%)	.514
AAD use (class I/III)				
Pre-procedure	77 (89.5%)	17 (89.5%)	60 (89.6%)	
Post-procedure	54 (62.8%)	13 (68.4%)	41 (61.2%)	.759
Net change in AAD use	−26.7%	−21.1%	−28.4%	
Complications^a^	3 (3.5%)	1 (5.3%)	2 (3.0%)	.532

*Abbreviations:* AAD, anti-arrhythmic drug; PVC, premature ventricular contraction; RMN, robotic magnetic navigation. ^a^Complications: manual group—one tamponade; RMN group—one pericardial effusion and one pseudoaneurysm. Symptom resolution (complete resolution of PVC-related symptoms) was achieved in 62.5% of the manual group and 74.6% of the robotic group (*P* = .514), showing no statistically significant difference. The remaining patients had either persistent symptoms or missing values for symptom resolution in the data. Post-procedure AAD use was 68.4% in the manual group versus 61.2% in the robotic group (*P* = .759). Both groups demonstrated reductions in AAD use from baseline, with the manual group showing a 21.1% net reduction and the robotic group showing a 28.4% net reduction. Three total complications occurred.

Class I/III AAD use was frequent at baseline in both groups (89.5% overall; 17/19 [89.5%] manual vs. 60/67 [89.6%] RMN). At 1 month, AAD use had declined to 62.8% overall (54/86), with similar proportions in manual and RMN cohorts (68.4% [13/19] vs. 61.2% [41/67]; *P* = .759). The net reduction in AAD use from baseline to 1 month was −21.1% in the manual group and −28.4% in the RMN group (overall, −26.7%), suggesting a numerically greater, but purely descriptive and not statistically compared, reduction in the RMN cohort.

Three periprocedural complications (3.5%) were recorded: one tamponade in the manual group and two events in the RMN group (one pericardial effusion and one pseudoaneurysm). Complication rates did not differ significantly between manual and RMN ablation (5.3% vs. 3.0%; *P* = .532). No additional major adverse events were identified.

### Procedural and clinical outcomes by premature ventricular contraction location

Procedural and short-term outcomes stratified by PVC location are shown in **Table 4**. The most common sites of origin were the RVOT (n = 22) and LVOT (n = 16). CS/epicardial PVCs (n = 15) were exclusively treated with RMN, whereas other locations, including papillary muscle (n = 10), combined LVOT/RVOT (n = 7), other LV free wall (n = 6), fascicular/septal (n = 5), and mitral annulus (n = 5), included both manual and RMN cases.

**Table 4:** Procedural Outcomes by PVC Location

PVC Location	N (M/R)	Procedure Time, min			Fluoroscopy Time, min			Symptom Resolution	Complications
		Manual	RMN	*P* Value	Manual	RMN	*P* Value		
RVOT	22 (7/15)	180.4 ± 60.2	215.4 ± 78.7	.535	14.4 ± 13.1	10.5 ± 9.1	.630	14/20 (70.0%)	1
LVOT	16 (3/13)	253.3 ± 105.6	185.5 ± 71.2	.189	12.8 ± 10.3	10.7 ± 2.6	.590	12/16 (75.0%)	0
CS/epicardial	15 (0/15)	—	242.3 ± 47.7	—	—	13.6 ± 5.5	—	8/13 (61.5%)	1
Papillary muscle	10 (2/8)	298.5 ± 116.7	181.9 ± 76.8	—	12.2 ± 8.3	16.4 ± 13.5	—	6/9 (66.7%)	1
LVOT/RVOT	7 (2/5)	229.0 ± 21.2	240.6 ± 50.4	—	11.2 ± 2.8	13.3 ± 2.1	—	6/6 (100.0%)	0
Other LV free wall	6 (3/3)	239.3 ± 67.7	224.3 ± 25.0	.700	11.7 ± 4.1	9.8 ± 0.7	.825	6/6 (100.0%)	0
Fascicular/septal	5 (1/4)	147.0 ± NA	211.0 ± 21.7	—	6.8 ± NA	15.5 ± 6.6	—	1/4 (25.0%)	0
MV annulus	5 (1/4)	208.0 ± NA	192.2 ± 97.5	—	8.0 ± NA	9.1 ± 2.3	—	4/5 (80.0%)	0

*Abbreviations:* CS, coronary sinus; LV, left ventricular; LVOT, left ventricular outflow tract; M, manual; MV, mitral valve; NA, not available; PVC, premature ventricular contraction; R, RMN; RMN, robotic magnetic navigation; RVOT, right ventricular outflow tract; SD, standard deviation. This table presents location-specific comparisons of procedural times between manual and RMN techniques. CS/epicardial cases (n = 15) were performed exclusively with robotic navigation. For locations where both techniques were used with adequate sample sizes for comparison (RVOT, LVOT, and other LV free wall), no statistically significant differences in procedure or fluoroscopy times were observed between techniques. Statistical comparisons were not performed for locations with small sample sizes (*n* < 3 in either group). Symptom resolution and complication rates are shown for each location, regardless of technique. Values are shown as mean ± SD for procedure/fluoroscopy times or n/N (%) for symptom resolution. *P* values for times were calculated using the Wilcoxon rank-sum test and not calculated for comparisons with n < 3 in either group.

Across PVC locations, there were no statistically significant differences in procedure time (*P* = .334), fluoroscopy time (*P* = .349), symptom resolution (*P* = .178), and complication rates (*P* = .855). Symptom-resolution rates varied from 25.0% for fascicular/septal PVCs to 100% for LVOT/RVOT and other LV free wall PVCs, but these comparisons were limited by small numbers within each category. Collectively, the data suggest broadly similar procedural efficiency and short-term clinical outcomes across diverse PVC substrates, with RMN preferentially employed for CS and epicardial cases.

### Characteristics of cases converted from robotic magnetic navigation to manual

Seven patients required intraprocedural conversion from RMN to manual ablation **(Table 5)**. These cases involved PVCs arising from a range of locations, including the LV anteroseptal wall, distal CS, RVOT, LVOT-CS, LVOT, and RV base. The mean procedure and fluoroscopy times in these converted cases were generally within the ranges observed for the main cohorts, reflecting increased complexity rather than outright procedural failure with RMN alone.

**Table 5:** Characteristics and Outcomes of Cases Converted from RMN to Manual Ablation

Patient	PVC Location	Age, years	LVEF, %	Pre-PVC, %	Post-PVC, %	Procedure Time, min	Fluoroscopy Time, min	Symptoms	AAD Pre → Post	Complications
13	LV anteroseptal	70	38	—	—	266	48.3	Resolved	Yes → Yes	None
20	Distal CS	56	50	25.4	—	321	50.4	Not fully resolved	No → Yes	Hematoma
23	RVOT	54	65	20.0	—	180	8.4	Resolved	Yes → No	None
41	RV anterior wall	62	45	16.8	0.7	352	5.2	Resolved	Yes → No	None
53	LVOT-CS	70	55	16.0	—	207	34.5	Resolved	Yes → No	None
62	LVOT	84	60	12.0	—	260	13.5	Resolved	Yes → Yes	None
89	RV base	39	—	10.7	—	257	8.3	Resolved	Yes → No	None

*Abbreviations:* AAD, anti-arrhythmic drug; CS, coronary sinus; LV, left ventricular; LVEF, left ventricular ejection fraction; LVOT, left ventricular outflow tract; PVC, premature ventricular contraction; RMN, remote magnetic navigation; RV, right ventricular; RVOT, right ventricular outflow tract. This table details the seven patients who required intraprocedural conversion from robotic to manual ablation to better understand which cases may be challenging for robotic navigation. These patients underwent successful manual ablation following conversion, with varied outcomes in symptom resolution and AAD requirements.

Most converted patients ultimately achieved symptom resolution after manual completion, and several were able to discontinue AAD therapy (eg, multiple yes-to-no transitions in AAD use). One patient experienced a hematoma; no tamponade or life-threatening complications occurred in this subset. These cases illustrate specific scenarios in which RMN was judged insufficient to complete the procedure but manual ablation successfully salvaged the case. Because they were excluded from the primary RMN cohort, the main per-protocol comparison may somewhat underestimate the challenges associated with an initial RMN strategy.

### Subgroup analysis by left ventricular systolic function (left ventricular ejection fraction <50%)

Subgroup analyses by LV systolic function (LVEF ≥50% vs. <50%) are presented in **Table 6**. Among patients with preserved LV systolic function (LVEF ≥ 50%; n = 46; 13 manual and 33 RMN), symptom resolution at 1 month was achieved in 72.7% (8/11) of manual patients and 81.2% (26/32) of RMN patients (*P* = .672). Procedure times were similar between manual and RMN in this subgroup (215.2 ± 55.4 vs. 217.2 ± 69.6 min; *P* = .916). Net AAD use declined in both groups, with a larger decrease in the RMN cohort (−30.8% manual vs. −48.5% RMN).

**Table 6:** Subgroup Analysis Comparing Outcomes Between Patients with and Without LV Systolic Dysfunction (LVEF < 50%)

Outcome	Value
No LV systolic dysfunction	
N (manual/RMN)	46 (13/33)
Symptom resolution	
Manual	8/11 (72.7%)
RMN	26/32 (81.2%)
*P* value	.672
Procedure time, min	
Manual	215.2 ± 55.4
RMN	217.2 ± 69.6
*P* value	.916
Net AAD change	
Manual	−30.8%
RMN	−48.5%
LV systolic dysfunction	
N (manual/RMN)	36 (6/30)
Symptom resolution	
Manual	2/5 (40.0%)
RMN	17/27 (63.0%)
*P* value	.374
Procedure time, min	
Manual	225.7 ± 112.6
RMN	207.7 ± 64.5
*P* value	.718
Net AAD change	
Manual	0.0%
RMN	−6.7%

*Abbreviations:* AAD, anti-arrhythmic drug; LV, left ventricular; LVEF, left ventricular ejection fraction; RMN, robotic magnetic navigation. In patients without LV systolic dysfunction (n = 46), symptom resolution rates were similar between the manual (72.7%) and robotic (81.2%) groups (*P* = .672), with both groups showing substantial reductions in AAD use (−30.8% and −48.5%, respectively). Among patients with LV systolic dysfunction (n = 36), the robotic group demonstrated higher symptom resolution (63.0%) compared to the manual group (40.0%), though this difference was not statistically significant (*P* = .374). Also among patients with LV systolic dysfunction, the manual group showed no net change in AAD use (0.0%), while the robotic group achieved a 6.7% reduction, though the small sample size (particularly n = 6 in the manual group) limits definitive conclusions. LV systolic dysfunction was defined as LVEF < 50%. *P* values compare manual versus RMN within each subgroup.

In patients with LV systolic dysfunction (LVEF < 50%; n = 36; 6 manual and 30 RMN), symptom resolution was observed in 40.0% (2/5) of manual cases and 63.0% (17/27) of RMN cases (*P* = .374). Procedure times again did not differ significantly (225.7 ± 112.6 vs. 207.7 ± 64.5 min; *P* = .718). Net AAD use was unchanged in the manual group (0.0%) and modestly reduced in the RMN group (−6.7%).

### Intention-to-treat sensitivity analysis

In the ITT sensitivity analysis **(Table 7)**, patients were grouped according to initial ablation strategy (RMN-initial, n = 74; manual-initial, n = 19), with the seven conversion cases retained in the RMN-initial cohort. Procedural metrics remained similar between groups. The mean procedure time was 217.1 ± 67.9 min in the RMN-initial group and 218.5 ± 74.7 min in the manual-initial group (*P* = .942). The mean fluoroscopy time was 13.5 ± 9.6 versus 12.4 ± 9.0 min, respectively (*P* = .665).

**Table 7:** ITT Comparison of Patients Grouped by Initial Ablation Strategy

Outcome	RMN-initial (n = 74)	Manual-initial (n = 19)	*P* Value
Procedure time, min	217.1 ± 67.9	218.5 ± 74.7	.942
Fluoroscopy time, min	13.5 ± 9.6	12.4 ± 9.0	.665
Symptom resolution at 1 month			.349
Resolved	53/74 (71.6%)	10/19 (52.6%)	
Not fully resolved	17/74 (23.0%)	6/19 (31.6%)	
Missing	4 (5.4%)	3 (15.8%)	
AAD use (class I/III)			
Baseline	66/74 (89.2%)	17/19 (89.5%)	
1 month post-procedure	44/74 (59.5%)	13/19 (68.4%)	.600
Periprocedural complications	3/74 (4.1%)	1/19 (5.3%)	1.000

*Abbreviations:* AAD, anti-arrhythmic drug; ITT, intention to treat; RMN, robotic magnetic navigation. All cases initially assigned to RMN, including seven procedures that converted to manual ablation, are analyzed in the RMN-initial group and compared with manual-initial cases. Procedural times, fluoroscopy times, 1-month symptom resolution, class I/III AAD use, and periprocedural complications were similar between groups, with no statistically significant differences in any endpoint. Cases were grouped by initial ablation strategy (RMN-initial includes seven cases that were converted to manual). *P* values from Student’s *t* test for continuous variables and Fisher’s exact test for categorical variables are given.

Among patients with available 1-month symptom data, clinician-documented complete symptom resolution occurred in 71.6% (53/74) of RMN-initial patients and 52.6% (10/19) of manual-initial patients, while 23.0% (17/74) and 31.6% (6/19) had persistent or recurrent symptoms (*P* = .349). Symptom status was missing in four RMN-initial and three manual-initial patients.

Class I/III AAD use at baseline was frequent in both ITT groups (89.2% [66/74] RMN-initial and 89.5% [17/19] manual-initial). At 1 month, AAD use declined to 59.5% (44/74) in the RMN-initial group and 68.4% (13/19) in the manual-initial group (*P* = .600), reflecting similar net reductions in AAD use without a statistically significant between-group difference. Periprocedural complications remained comparable between strategies (4.1% [3/74] RMN-initial vs. 5.3% [1/19] manual-initial; *P* = 1.000). These ITT findings were consistent with the primary per-protocol results and did not reveal any statistically significant differences in procedural performance, early symptom relief, AAD use, or complication rates according to initial ablation strategy.

## Discussion

In this single-center retrospective cohort, RMN-guided catheter ablation for frequent PVCs achieved procedural efficiency and very short-term clinical outcomes comparable to conventional manual ablation. Total procedure time and fluoroscopy time were similar between strategies, with mean case durations of approximately 3.5 h and mean fluoroscopy times near 12 min in both groups. At approximately 1 month, clinician-documented symptom resolution was observed in roughly three-quarters of RMN patients and about two-thirds of manual patients, without a statistically significant difference, and class I/III AAD use declined substantially in both cohorts. Overall complication rates were comparable. Because we did not systematically collect post-ablation PVC burden or follow-up LV function, these findings primarily inform procedural performance and early, practice pattern–dependent outcomes and should be interpreted as hypothesis-generating support for RMN as a feasible alternative to manual PVC ablation across a broad range of substrates in routine practice.^4–11,19–21^

Importantly, the absence of statistically significant differences in procedure time, fluoroscopy exposure, 1-month symptom resolution, AAD use, or complication rates should not be interpreted as evidence of equivalence between RMN and manual ablation. The overall cohort, and particularly the manual arm, was relatively small, and the study was not powered to exclude modest differences between strategies. Accordingly, our results should be viewed as hypothesis-generating and as providing preliminary comparative data rather than definitive proof of noninferiority or equivalence. Prior observational and prospective data have shown that RMN can provide at least similar acute success and safety compared with manual ablation for ventricular and supraventricular arrhythmias, often with meaningful reductions in fluoroscopy exposure.^12–18^ In contrast to some earlier series in which RMN procedures were longer during the learning phase,^12–14,18^ we observed no prolongation of procedure time relative to manual ablation, suggesting that, by the study period, the operators had largely overcome the learning curve. At the same time, fluoroscopy times were already low in both arms, reflecting contemporary mapping-based workflows. In this context, RMN did not further reduce patient fluoroscopy time but would be expected to substantially reduce operator exposure, which was not directly measured in this study.^12–18^

Both cohorts had a high prevalence of class I/III AAD use at baseline (nearly 90% of patients), consistent with referral for ablation after failed or poorly tolerated drug therapy.^10,11^ After ablation, the proportion of patients on AADs declined to about two-thirds overall, with a numerically greater reduction in the RMN group than in the manual group (−28.4% vs. −21.1%). Although our study was underpowered to detect modest between-group differences and did not include formal statistical testing of the change in class I/III AAD use from baseline, these descriptive findings suggest that successful PVC ablation, whether manual or RMN-guided, can allow de-escalation of AAD therapy in many patients.^4–8,11,19–21^ Any potential incremental advantage of RMN in facilitating AAD discontinuation remains speculative and may also reflect local practice patterns and physician comfort. Therefore, these observations should be regarded as hypothesis-generating rather than as evidence of superiority.

PVC origins in this cohort included common outflow tract sites as well as more anatomically complex locations such as papillary muscles, CS-adjacent targets, and epicardial regions.^9–11^ RMN was used exclusively for CS/epicardial cases (n = 15), whereas outflow tract and papillary muscle sites were treated with either approach.^19–21^ Because CS/epicardial cases are typically more time-intensive and occurred only in the RMN arm, we performed a sensitivity analysis excluding these cases from procedural time comparisons; subsequent results were unchanged from the primary analysis **(Table S1)**.^19–21^ Across locations with adequate sample size for comparison, procedure time, fluoroscopy time, and short-term symptom resolution did not differ significantly between techniques. Collectively, these descriptive data suggest that both manual and RMN strategies can be used effectively across a range of PVC substrates, with RMN more often selected for complex or epicardial-adjacent targets where stability and directional control may be advantageous.^9–11,14–16,19–21^

A small proportion of cases initially attempted with RMN required intraprocedural conversion to manual manipulation. These conversions—approximately 1 in 10 of all RMN-attempted cases—spanned diverse anatomic locations and generally had procedure and fluoroscopy times within the range of the primary cohorts. Most converted patients ultimately achieved symptom resolution, and several were able to discontinue AAD therapy. From a practical standpoint, these conversions underscore that RMN has technical limitations and that manual catheter manipulation remains an important complementary strategy. Although conversion triggers were not prospectively standardized, conversion generally occurred when adequate reach and stable contact at the intended target could not be maintained with the magnetic catheter, prompting completion of ablation with manual manipulation.^12–16,19–21^ In our primary per-protocol analysis, converted cases were analyzed separately and excluded from the RMN cohort, which isolates outcomes of “pure” RMN and manual procedures. However, an ITT sensitivity analysis that grouped converted cases with the RMN-initial cohort yielded procedural metrics, early symptom relief, AAD use, and complication rates that were very similar to those observed in the per-protocol comparison and showed no statistically significant differences between initial strategies. Taken together, these findings suggest that, while conversions reflect the real-world limitations of RMN, their inclusion does not materially alter the overall comparative picture in this modest single-center cohort and that larger studies will be needed to identify predictors of conversion and clarify its impact on outcomes.^19–21^

We observed one tamponade in the manual arm and one pericardial effusion plus one vascular pseudoaneurysm in the RMN arm, with no significant difference in event rates between the groups. These complication rates are consistent with published series of PVC ablation and support the safety of both techniques in experienced centers.^4–9,11,19–21^ Importantly, the use of RMN did not introduce any unique complication patterns in this cohort. Although operator radiation exposure and musculoskeletal strain are important considerations that favor RMN conceptually, these were not captured in our dataset and warrant formal evaluation in future prospective studies.^12–18^

This study has several limitations. First, it is a retrospective, nonrandomized, single-center analysis with a relatively small manual ablation cohort, which limits the power to detect modest between-group differences and introduces potential selection bias. Technique choice was at the discretion of the operator and may have been influenced by PVC location, perceived complexity, or system availability, factors that could confound comparisons. Second, our primary comparison of RMN versus manual ablation was per-protocol, excluding cases that converted from RMN to manual in order to maintain clearly defined “pure” RMN and manual groups. We addressed this by performing an ITT sensitivity analysis that grouped all RMN-initial cases, including conversions, together. The ITT results were concordant with the per-protocol findings, but both analyses remain underpowered and nonrandomized. Third, follow-up was limited to approximately 1 month, focusing on symptom resolution and AAD use. We did not systematically capture long-term PVC burden, recurrence rates, or recovery of LV function in patients with suspected PVC-induced cardiomyopathy,^1–9,21^ so our outcome assessment is restricted to very short-term, largely subjective, and practice pattern–dependent endpoints rather than objective, longitudinal measures of ablation efficacy. Fourth, objective measures of operator experience, learning-curve effects, radiation dose, and cost were not available, despite prior data suggesting that RMN may reduce operator radiation exposure and musculoskeletal strain compared with manual ablation.^12–18^ Finally, subgroup analyses by PVC location and LV systolic function (LVEF < 50%) were underpowered and should be viewed as descriptive rather than confirmatory.

## Conclusions

In this single-center retrospective cohort of patients undergoing catheter ablation for frequent PVCs, RMN achieved procedural times, fluoroscopy times, and short-term symptom outcomes that were comparable to those of conventional manual ablation. Both approaches were associated with substantial reductions in class I/III AAD use at 1 month. The reduction was numerically greater in the robotic group, but this difference was descriptive and not formally tested, and overall complication rates were similar between the techniques. These findings, which were consistent in an ITT sensitivity analysis, support RMN as a feasible alternative to manual PVC ablation across a broad range of substrates and highlight the need for larger, multicenter studies with longer follow-up, systematic rhythm monitoring, and operator-centric endpoints to better define the optimal role of robotic systems in PVC management.
